# How does the genomic naive public perceive whole genomic testing for health purposes? A scoping review

**DOI:** 10.1038/s41431-022-01208-5

**Published:** 2022-10-19

**Authors:** Isabella A. Sherburn, Keri Finlay, Stephanie Best

**Affiliations:** 1 Australian Genomics, Murdoch Children’s Research Institute, Melbourne, VIC Australia; 2 Department of Health Services Research, Peter MacCallum Cancer Centre, Melbourne, VIC Australia; 3 Victorian Comprehensive Cancer Centre, Melbourne, VIC Australia; 4 Sir Peter MacCallum Cancer Centre Dept of Oncology, University of Melbourne, Melbourne, VIC Australia

**Keywords:** Social sciences, Medical genomics

## Abstract

The benefits of genomic testing are primarily reported in rare disease, cancer diagnosis and disease management. However, as research into its application in common, more complex conditions grows, as well as the increased prevalence of carrier screening programs, the genomic naive public is more likely to be offered testing in future. To promote social acceptability and ethical application of this technology, it is essential that public perceptions of genomics are considered. Previous studies, however, have primarily focussed on the views of those with genetic conditions or those undergoing genetic testing. The aim of this scoping review is to investigate the genomic naive public’s perceptions of clinical genomics and clinical genomic testing. Embase, MEDLINE and PubMed databases were searched, with a total of 3460 articles identified. Data analysis was organised according to the nonadoption, abandonment, scale-up, spread, and sustainability (NASSS) framework. Sixteen full-text articles were included in the final analysis. Most of the studies used questionnaires to determine attitudes of the public toward clinical genomics (*n* = 12). Public perceptions were found to underpin technology (Domain 2), value proposition (Domain 3), the adopter system (Domain 4) and the wider context (Domain 6) of the NASSS framework, highlighting its importance when considering implementation of an innovative technology such as genomic testing. Our study shows public perceptions are diverse, and highlights the need for more studies on the views of underrepresented groups and the impact of cultural contexts on perceptions.

## Introduction

Whole genome sequencing (referred to as genomic testing from hereon in) has been previously limited to research environments, however it has led to improved diagnostic rates and management in healthcare settings for patients with rare disease or cancer [1–3]. There is increasing research into its potential impact on complex conditions (e.g. diabetes, neurodegenerative disorders) [1] and its usefulness in carrier screening [4–6], meaning it is likely that the wider public will be offered genomic testing in routine healthcare practice in future. Genomic testing, however, has many ethical and practical considerations which can impact its implementation. Despite the complexity of genomic testing, several studies have focussed solely on the return of specific types of results from genomic testing [3, 7–11]. Additionally, these complexities can be perceived differently depending on whether an individual is symptomatic or asymptomatic [12].

Unique issues related to genomic testing are the identification of incidental findings (IFs) and variants of unknown significance (VUSs) [12, 13]. IFs are gene variants that are found during genomic testing but are unrelated to the condition or symptoms being investigated [12–14]. IFs pose several challenges including difficulties in consistent reporting of medically actionable findings, and their potential impact on biological relatives [12, 13]. As genomic testing investigates the entire genome rather than a specific set of genes, it is more likely an IF will be found [12, 13]. A literature review conducted by Delanne et al. reported that many participants wanted to be involved in the variant selection process [3]. Another study found that adolescents who undergo testing would also like to be involved in this process [10], demonstrating the need to consider the public when implementing genomic testing at a population level.

A VUS is a genetic variant with unknown pathogenicity [12]. Challenges with VUS identification include: changes in categorisation of variants as research advances, time-consuming functional studies may be required if wanting to re-categorise variants to pathogenic, and uncertainty exacerbating stress in patients [12]. Genomic testing can identify VUSs spanning under-researched areas of the genome—a challenge not as prominent in single gene testing or exome analysis [12]. A US study found that participants were less likely to want to know about VUSs compared to IFs, most likely due to the non-actionable nature of VUSs [9]. Similarly, Delanne et al. found that in studies where actionable and non-actionable findings were discerned, there was generally more acceptance of actionable findings [3]. While a VUS may be perceived as a potential answer for symptoms for someone with a rare disease and therefore more acceptable, someone without any current symptoms this may cause unnecessary anxiety.

Many studies exploring the use of genomic testing have focussed on the perceptions of those with a specific condition e.g., in the cancer, genetic, undiagnosed, and rare disease community, who are more likely to be offered genetic and genomic testing due to the high clinical utility [15]. For example, Boardman and colleagues have conducted several studies with people with spinal muscular atrophy and their thoughts on carrier testing for their condition [16, 17]. Views of those with other genetic conditions have also been researched [17, 18]. However, many members of the public are already undertaking recreational genomic testing via online companies (e.g. Ancestry.com, 23AndMe) [19–21] despite the utility of health findings being low or even negligible [22, 23].

Although there has been research into the perspectives of the asymptomatic population undertaking recreational genomic testing [19–21], their perception of clinical genomic testing has not been well-addressed. Genomic testing in the clinic for asymptomatic individuals could include carrier screening, where a specific genetic variant is investigated [4], or newborn screening (NBS), where a specific set of metabolic conditions are investigated [5, 6]. These types of tests have more clinical utility and are more likely to be offered to the general population. However, genomic testing expands carrier testing possibilities as more variants can be identified [4–6]. For example, NBS is a form of testing with high participation across Western countries [24, 25], however genomic testing can allow for the identification of conditions where there is no current treatment. DeLuca found that US participants were generally in favour of expanded NBS but acceptance towards testing for conditions without treatment was lower [26]. The BabySeq project furthers this research by assessing the impact of sequencing a newborn’s genome for future conditions and using the information to prevent onset [27]. The introduction of this trial amongst the general public reinforces the need to identify the public’s perceptions of genomic testing.

Aside from differences between the genetic, undiagnosed, and rare disease community and the general population, there are also many benefits to considering the public’s views in research studies including improved quality of research and ensuring it is relevant to the community [28]. This can increase social acceptability of genomic testing and promote its safe and appropriate implementation into healthcare [28]. Therefore, the research questions we aim to address in this scoping review are (a) What studies have been undertaken to discern the public’s perceptions of genomic testing? and (b) How can public perceptions inform implementation of genomic testing more broadly using the NASSS framework?

## Methods

A scoping review was used to provide an overview of the current literature available, describe how the research is conducted, identify key concepts discussed, and any gaps in the literature [29, 30]. This approach is appropriate as our research question has not been explored before and there is minimal research in the area.

### Search strategy

The literature search was conducted from January 2010 to August 2022 in line with the Preferred Reporting Items for Systematic reviews and Meta-Analyses extension for Scoping Reviews (PRISMA-ScR) Checklist [31] (Supplementary File 1). The PRISMA-ScR was also used to guide reporting of this review. The year 2010 was chosen as the earliest publication date because the field of genomics has progressed rapidly with improved technology [32]. It is also noted that public perceptions of genetic testing can change with time [33] and more recent data would be most informative for our research question.

We took advice from a specialist librarian to guide the search strategy (Supplementary File 2). The databases Embase, MEDLINE and PubMed were interrogated. Search terms were chosen through an exploration of Medical Subject Headings (MeSH) terms and consideration of key words in current articles related to public perceptions of clinical genomics. We define ‘clinical genomics’ here as any genomic test that can be used to diagnose a health condition within a clinical setting, rather than a research setting (i.e. sequencing genomes for the purposes of population studies). Search terms included related to the general public, perceptions and genomic testing. Titles and abstracts containing the terms “direct-to-consumer testing”, “personal genomic testing”, “ancestry or genealogy testing” or “recreational genomics” were excluded from the search as clinical genomics was the focus. Articles were downloaded into Endnote X9 [34], a bibliographic database. Duplicates and incomplete references were discarded resulting in 3460 unique peer reviewed articles for screening.

### Selection of studies

Rayyan (https://www.rayyan.ai/), a web-based application [35], was used to facilitate independent screening of articles by two reviewers (SB, IS). A third reviewer (KF) assisted in discussing articles that caused disagreement. Sets of 50 articles were independently screened by two reviewers (SB, IS) until an inter-rater reliability score of 0.75 (i.e. substantial agreement) was achieved [36]. This process allowed for refinement of the inclusion and exclusion criteria. One reviewer (IS) screened the remaining titles and abstracts using the finalised inclusion and exclusion criteria (Table 1) with weekly meetings to discuss decisions.

**Table 1:** Inclusion and exclusion criteria.

Inclusions:
• Genomic naive individuals (i.e. those who do not have a known heritable/genetic condition, or a first- or second-degree relative with a heritable/genetic condition and are not well-informed on genomics)
• Individuals that have not been offered or exposed to genetic services (aside from newborn screening and cancer screening as these are undertaken in mainstream settings) e.g. genetic counselling, genetic/genomic testing
• If the study focussed on the clinical application of genomic testing

Exclusions:
• If the individual has a heritable/genetic condition
• If the individual has a first- or second-degree relative with a heritable/genetic condition
• If the individual has received/undertaken any kind of genetic service (e.g. genetic counselling, genetic/genomic testing). This excludes newborn and cancer screening as these are undertaken in mainstream settings
• The study is excluded if it focuses on a genomic test for a specific genetic condition (e.g. BRCA testing)
• If the study focussed on personal or recreational genomic testing
• If the study focussed on single gene testing
• If the study solely focussed on willingness to partake in genomic research

A key inclusion item was the ‘genomic naive’ public. We define the term as an individual that has not had genetic or genomic testing and does not have a known family history of a genetic disease which requires rigorous preventative strategies for asymptomatic individuals. This definition was decided upon through an iterative process of assessing the study populations in articles we deemed relevant to our research question. However, it is acknowledged that genomic literacy exists on a continuum [37].

**Fig. 1:**
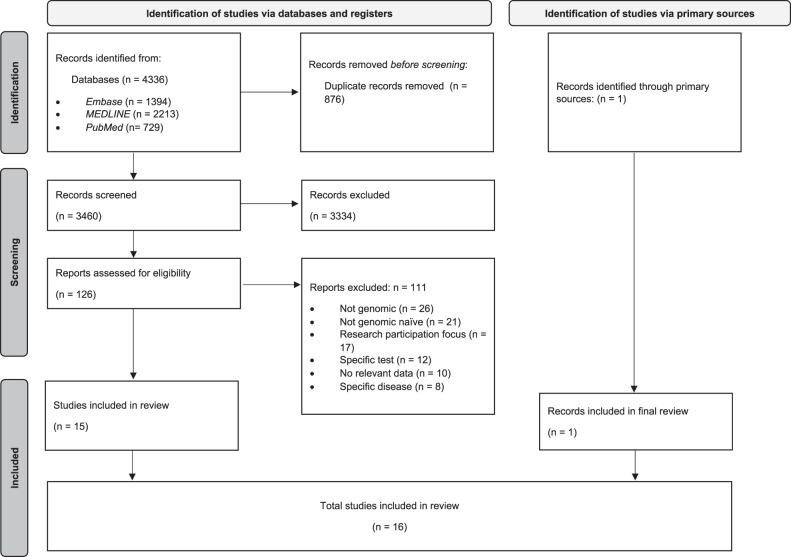
PRISMA-ScR [31] flow diagram. 3460 unique records were identified through an electronic search of Embase, Medline and PubMed, of which 126 proceeded to full-text review. An additional article was retrieved through the primary sources. A total of 16 studies were included in the review.

The resultant full text articles (*n* = 126) were screened by two reviewers (SB, IS), with the first 20 reviewed independently to ascertain agreement. One reviewer (IS) screened the remaining full text articles with regular review meetings. Reasons for exclusion are noted in Fig. 1. An additional article was discovered through mining of full text article references and included in the final analysis. The final 16 full-text articles were then analysed. The PRISMA [38] flowchart is shown in Fig. 1.

### Data extraction and analysis

The final 16 full text articles were re-read by two reviewers (IS and SB) to determine categories for data extraction. An Excel spreadsheet was used to record all data extracted. Descriptive data gathered from the included studies were: author, year published, aim of paper, qualitative vs quantitative study, and primary type of respondent. Key findings were also recorded.

Data analysis was conducted using the nonadoption, abandonment, scale-up, spread, and sustainability (NASSS) framework developed by Greenhalgh et al. [39, 40]. The framework was developed recently and has been used to examine various technology-based health interventions e.g., teleconsultation implementation [41, 42] and several e-health tools [43–45]. This framework was deemed appropriated as one of its key purposes is to plan the implementation, scale-up or rollout of technological innovations in healthcare [39]. The framework domains include: Domain 1 (Health condition), Domain 2 (Technology), Domain 3 (Value proposition), Domain 4 (Adopter System), Domain 5 (Healthcare organisation), Domain 6 (Wider institutional and social context), and Domain 7 (Embedding and adaptations over time) [39]. Each domain has several ‘questions’ for researchers to consider [39, 40] (Table 2). Key findings from the primary sources were mapped to the relevant domains. These domains were also used to structure reporting of results. Analysis was completed by one reviewer (IS) who had regular meetings with another reviewer (SB) to discuss any challenges.

**Table 2:** Greenhalgh et al’s nonadoption, abandonment, scale-up, spread, and sustainability (NASSS) [34, 35] Framework Domains.

Domain name	Domain questions
Domain 1: Health condition	1A: Nature of condition or illness
	1B: Comorbidities
	1C: Socio-cultural factors
Domain 2: Technology	2A: Material properties
	2B: Knowledge to use
	2C: Knowledge generated
	2D: Supply model
	2E: Who owns the IP?
Domain 3: Value proposition	3A: Supply-side value (to developer)
	3B: Demand-side value (to patient)
Domain 4: Adopter System	4A: Staff (role, identity)
	4B: Patient (passive vs active input)
	4C: Carers (available, type of input)
Domain 5: Healthcare organisation	5A: Capacity to innovate
	5B: Readiness for this technology
	5C: Nature of adoption/funding decision
	5D: Extent of change needed to organisational routines
	5E: Work needed to implement change
Domain 6: Wider institutional and social context	6A: Political/policy context
	6B: Regulatory/legal issues
	6C: Professional bodies
	6D: Socio-cultural context
	6E: Inter-organisational networking
Domain 7: Embedding and adaptations over time	7A: Scope for adaptation over time
	7B: Organisational resilience

## Results

### Study characteristics

Sixteen studies were included in the analysis [46–61]. Most were quantitative (*n* = 12), using questionnaires to assess public perceptions [46–52, 54, 56, 57, 59, 61]. Three studies conducted focus groups [53, 55, 60] while one study used both focus groups and a survey [58]. The US has contributed the most to this field thus far, undertaking six of the 16 studies identified in the literature search [49, 50, 52, 53, 55, 58]. This is followed by Canada (*n* = 2) [48, 51] and Japan (*n* = 2) [54, 59]. Each of the following countries contributed one study: Jordan [56], Korea [57], The Netherlands [61], Singapore [60], Qatar [46] and the UK [47]. Ten of the studies attempted to recruit a representative sample [46–49, 51, 53, 54, 56, 59, 61]. Higher educated participant populations (compared to the general population) were noted in four studies [48, 59–61]. Three studies recruited participants from specific sites [52, 55, 57]. No studies attempted to discern the views of underrepresented populations aside Mallow et al. [58] who conducted focus groups with a rural community (Table 3, Supplementary File 3).

**Table 3:** Study characteristics.

Author	Country	Aim	Sample size (*n*)	Recruitment strategy	Representative population	Data collection strategy
Abdul Rahim et al. [46]	Qatar	To measure public awareness and attitudes towards genetic and genomic testing in Qatari adults.	834	Representative cell phone sample using a list-based dialling technique.	Representative	Survey with questions regarding respondent demographics, knowledge, attitudes and willingness to undertake genomic testing.
Ballard et al. [47]	UK	To evaluate opinions regarding genomic tests in general, if they have familial implications, and how incidental findings should be returned.	1954	Survey conducted via YouGov, a company who has access to a community of 6 million people worldwide. YouGov sent an email with a link to the survey to a sub-population (*n* = 2005) of their UK community.	Representative	Survey with questions regarding conceptualisation of genomic tests in healthcare, genomic information of relevance to relatives of the person being tested, and unexpected genomic information.
Bombard et al. [48]	Canada	To determine Canadians willingness to partake in expanded newborn screening compared to traditional newborn screening.	1213	Recruitment through an internet panel provided by Survey Sampling International, which hosts online panels to support market and academic research. A demographically diverse sample, reflective of the Canadian population by age, gender and region of residence, consistent with 2011 statistics Canada data were eligible to complete the questionnaire.	Not representative	Survey included items to build and assess knowledge and to measure selected attitudes and demographics. followed by quizzes, a discrete choice experiment and a reasoning exercise.
Dodson et al. [49]	USA	The aim of this study was to assess the baseline interest of the public in whole genome testing for oneself, parents’ interest in whole genome testing for their youngest children, and factors associated with such interest.	Total = 2144; Parents *n* = 1539; Non-parents *n* = 605	Cross-sectional internet-based survey of a nationally representative sample of the US population. To ensure adequate representation, parents [defined as having child(ren) 0–17 years old living in the household] and particular racial minorities including African-Americans and Hispanics were oversampled.	Not representative	Survey with questions regarding respondent demographics, interest in whole genome sequencing for themselves and their youngest children.
Edgar et al. [50]	USA	To have a better understanding of the specific genomic risk information adoptees are interested in learning.	Total = 341; Non-adoptees = 229; Adopted individuals = 112	Non-adopted individuals were recruited via Amazon Mechanical Turk, a crowdsourcing Internet platform. Adopted individuals were recruited through 14 organisations and Facebook groups for adoptees.	Non-adopted individuals were reported as representative due to the use of Amazon Mechanical Turk. Adopted individuals were not reported as representative.	Survey items included patient demographics, level of contact with biological relatives, knowledge of family history, interest in receiving elective genomic testing results, motivations for wanting testing, perceived utility of results, willingness to pay, and social and personal identity.
Etchegary et al. [51]	Canada	To contribute to the gap in the literature and provide descriptive, attitudinal data that can inform the integration of genomics into clinical care in ways that accord with the public who will ultimately use the service.	689	Online survey was administered on SurveyMonkey and paid advertising enabled advertising of the survey link on Facebook. Facebook advertised the survey link to all registered users in the province. Targeted advertising was implemented as needed (e.g., to underrepresented users in the rural health authorities). The survey link was also shared widely through the research team’s and public council’s personal and professional networks, communication channels of Memorial University, and the provincial health authorities.	Not reported	Survey items measured the following: interest in WGS and information preferred for sequencing decisions, interest in pharmacogenomic testing specifically, attitudes toward various features of genome sequencing, preferences for the return of incidental findings, opinions about the secondary use of genomic data, and demographic items.
Gibson et al. [52]	USA	To determine patient knowledge, interest and willingness-to-pay for pharmacogenomics testing in a community pharmacy.	27	Qualtrics survey was distributed to 7019 email addresses of patients and customers of an independent community pharmacy. The study site is a community pharmacy in Murfreesboro (TN, USA) that offers a wide variety of clinical services.	Not representative but not reported	Survey with four main parts: patient demographics, patient pharmacogenomics knowledge, patient interest in pharmacogenomic testing, willingness-to-pay for a pharmacogenomic testing service.
Hahn et al. [53]	USA	This paper addresses the first study done as part of the educational needs assessment for the community. Using focus group methodology, we assessed the community’s awareness and perception of genomic medicine and preferences regarding educational strategies and content. Baseline knowledge will be used to inform educational interventions.	121	County Health Department and Chamber of Commerce representatives were used as key informants to identify organizations within the County that provided a representative population in terms of gender, ethnicity, religion, age, and socioeconomic status. Churches, colleges, employers, social groups, local Veteran’s Administration, and Army National Guard units were contacted for inclusion.The study participants were recruited from a single urbanized community in the Southeastern United States whose collective perceptions cannot be projected onto other communities.	Representative for gender and ethnicity only	To assess the community’s awareness and perception of genomic medicine and preferences regarding educational strategies and content, an open-ended, semi-structured interviews.
Hishiyama et al. [54]	Japan	This study aims to elucidate the public attitude towards the handling of genetic information during research and general medicine in the Japanese adult population.	3000	The participants of the survey were 3000 people recruited from the Ordinary Citizens Panel consisting of people aged 20 to 69 years. The Panel belongs to the “Ordinary Citizens Market Forecasting System” managed by the Mitsubishi Research Institute.	Not representative but not reported	Survey with questions regarding respondent demographics, knowledge and perception of genetic information, preferences when receiving results, attitudes towards the handling of genetic information, and concerns about genetic testing.
Joseph et al. [55]	USA	To inform policy debates by examining the views, perspectives, and values of healthy pregnant women, and parents of children with primary immunodeficiency disorders about both traditional newborn screening and expanded newborn screening.	Total = 31; Pregnant women (*n* = 26); Parents of children with a primary immunodeficiency disorder (*n* = 5)	Pregnant women were receiving prenatal care at either of two urban California medical sites, an academic medical hospital and a public hospital.	Not representative	Focus groups to discuss initial impressions and concerns regarding the Californian NBS program. Participants were also provided with two case studies of expanded NBS to facilitate further discussion.
Khadir et al. [56]	Jordan	To assess the knowledge and attitude towards genetic testing of the Jordanian population in general. This study also evaluates the knowledge and attitude of patients with immune diseases and how it compares to that of the general Jordanian population.	1149	Distribution using different generic social media platforms dedicated for Jordan in addition to other platforms targeting different Jordanian cities to ensure better representation of different segments of the Jordanian population.	Not reported	Survey questions include: demographic characteristics, genetic knowledge, perceived knowledge of genetics and attitudes toward genetic testing.
Lee et al. [57]	Korea	To evaluate the awareness and attitude towards personalised medicine in Korean adults.	703	Survey was distributed to 706 adults who visited community pharmacies or public healthcare centers between December 31, 2012 and January 14, 2013. The 13 study sites were comprised of four pharmacies that primarily fill prescriptions for outpatients from general hospitals, seven community pharmacies providing over-the-counter medications and filling prescriptions from nearby local clinics, and two community healthcare centers. All sites were located in the Seoul metropolitan area.	Not reported	Survey with three domains: public knowledge/awareness of personalized medicine, public attitude toward personalized medicine, and public acceptance of integrated pharmacogenomic testing as part of the national health examination.
Mallow et al. [58]	USA	To assess genomic and epigenetic knowledge and beliefs in rural West Virginia, USA.	Survey: *n* = 68; Focus group: *n* = 93	The recruitment for each forum was directed by the Community Partnership Board and the research team. Approximately 100 personal invitations per community were sent to community leaders and lay persons 1–3 weeks in advance. In addition, flyers were hung in local areas (places of worship, grocery stores, community centers, etc.) The survey was distributed to focus group participants at the end of the session.	Not reported	The community led the discussion and focus groups usually lasted from 1.5 to 2 hours. Discussions were around genes and family health history. The survey contained basic demographic information and qualitative and quantitative questions on their knowledge of family health history, willingness to partake in genetic studies, and the perceived influence of environmental and lifestyle factors on hereditary condition.
Okita et al. [59]	Japan	To survey Japanese adults interests and concerns of whole genome testing, their willingness to partake in research, and the factors that influence their attitudes.	2399	Participants were registered as part-time survey assistants with Video Research Ltd., to which we outsourced part of the survey. The subjects comprised males and females aged 16 years or older who matched the age distribution of the Japanese population based on the Japanese government’s national population census in 2010.	Not representative	A survey of awareness around WGS and studies using WGS.
Ong et al. [60]	Singapore	To determine whether the Singapore population prefer the term ‘precision medicine’ or ‘personalised medicine’.	Total = 24; English-speaking = 11; Mandarin-speaking = 8; Malay-speaking = 5	Not reported	Not representative	Focus groups began with discussions on baseline understanding of ‘precision medicine’ and ‘personalised medicine’. After an education video, participants were asked again which term they preferred. Participants were then able to discuss other concerns or thoughts the video provoked.
Vermeulen et al. [61]	The Netherlands	To determine the Dutch public’s opinion towards preventive genomics, their attitudes towards genetic testing and family history-based risk assessment for common chronic conditions, and what influences these attitudes.	978	Consumer panel representative of the Dutch population.	Not representative	Survey with questions regarding respondent demographics, knowledge of genetic tests, purchase of commercial genetic tests.

*NBS* newborn screening; *WGS* whole genome sequencing.

### Demographic characteristics

Education level influenced decisions to hypothetically partake in genomic testing in different ways [49, 51, 56, 59, 61]. Three studies found that more educated individuals were more likely to be interested in testing [49, 56, 59], while two other studies found that being more educated led to more critical attitudes towards testing [51, 61]. One study found no association between education level and attitude towards testing [57]. Khadir, Al-Qerem and Jarrar [56] found that having a low perceived knowledge of genomic testing’s social consequences reduced the likelihood of having a reserved attitude. Abdul Rahim et al. [46] found genetic/genomic knowledge did not impact whether a participant would engage in testing.

The age of the participant was reported to influence decision making [49, 54, 56, 57, 59], with no consensus on attitudes of older versus younger adults. Lee et al. [57] found that older adults were more likely to approve of integrating personalised medicine testing into standard healthcare. Two other studies also found that older adults were slightly more interested in genomic testing [54, 56]. In contrast, Okita et al. [59] found that older adults were less willing to partake in genomic testing, while Dodson et al. [49] found no association between age and likeliness to have testing.

Abdul Rahim et al. [46] found that marital status was not significantly associated with willingness to partake in testing in Qatari adults, while Dodson et al. [49] found American participants planning to have children in the next five years had significantly increased interest in testing. Dodson et al. [49] was the only study to investigate whether ethnicity influenced decision-making, showing no association.

Okita et al. [59] assessed the influence of employment status on willingness to partake, reporting that students had significantly more positive attitudes towards testing compared to employed respondents. Bombard et al. [48] found that having an income of more than CAD$80,000 led to a 11-12% decrease in likeliness of believing parents have a responsibility to have their child tested via expanded NBS. No study assessed the impact of sex on attitude towards testing, however Lee et al. [57] found that sex did not significantly influence whether the participant had heard of personalised medicine.

### Analysis using the NASSS Framework

**Fig. 2:**
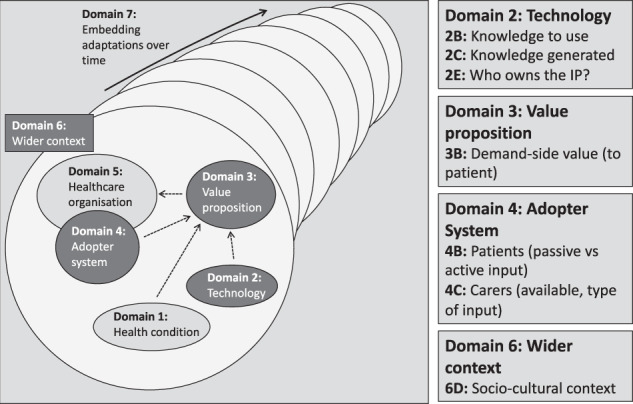
Adapted NASSS Framework [39, 40]. The NASSS Framework considers the influences on adoption, nonadoption, abandonment, spread, scale-up, and sustainability of healthcare technologies. Domains 2 (Technology), 3 (Value proposition), 4 (Adopter system) and 6 (Wider context) of the NASSS Framework have been addressed in this scoping review to consider how public perceptions are incorporated in the framework.

Using the NASSS domains we were able to map primary source data to technology (Domain 2), value proposition (Domain 3), the adopter system (Domain 4) and the wider context (Domain 6) (Fig. 2). Greenhalgh et al. [39] does not provide specific definitions for their domains, rather they frame these domains in the form of questions that need to be answered. We replicated this approach and adapted the questions to align with our study questions (Supplementary File 4).

### Domain 2: Technology

Domain 2 considers the technical aspects of the technology that will influence its implementation [39]. Questions 2B, types of data generated; 2C, knowledge needed to use the technology; and 2E, Who owns the IP generated by the technology?, are addressed in the primary sources.

#### Question 2B: Type of data generated

This question considers the knowledge generated by the technology and how this is perceived by patients and/or caregivers. Two studies cited the accuracy of genetic information as an issue for their participants [54, 58].

#### Question 2C: Knowledge needed to use the technology

Greenhalgh et al. [39] defines this as the type of knowledge needed by both healthcare providers and patients to use the technology. However, we will only focus on the views of the general public. Although patients of genomic testing do not necessarily need knowledge to undertake testing, the informed consent process is essential. To gain informed consent from patients, understanding the baseline genomic knowledge of the public is beneficial for those taking consent. Knowledge of genetics and genomics was assessed in several different ways across the included articles [46, 52–54, 56, 58, 60]. These included asking participants if they had heard of various genetic and/or genomic terms, how they had heard about genomic testing, how participants describe genomics (in a focus group setting) and questions on genetics knowledge.

Abdul Rahim et al. [46] found that less than a third (*n* = 245) of survey respondents had heard of genomic testing while just over half (*n* = 447) had heard of genetic testing. Gibson, Hohmeier and Smith [52] found that 54% (*n* = 7) of their participants had heard the term ‘pharmacogenomics’. Hishiyama, Minari and Suganuma [54] found that more than two-thirds of their participants had heard of classic genetic terminology (e.g. DNA, gene, chromosome), whereas fewer participants had heard of newer, genomics terminology (e.g. ‘personal genome’ and ‘pharmacogenomics’). Hahn et al. [53] found that the majority of their participants had not heard the term ‘genomic medicine’ and ‘personalised medicine’. Ong et al. [60] found that English and Mandarin-speaking participants had heard of the term ‘personalised medicine’ but not ‘precision medicine’, while Malay-speaking participants had not heard of either term.

Three studies questioned participants on how they had heard about genomics [46, 52, 53]. Abdul Rahim et al. [46] asked about both genetic and genomic testing whereas Gibson, Hohmeier and Smith [52] asked their participants where they had heard certain terms from. Abdul Rahim et al. [46] found that 30% (*n* = 69) of participants who knew of genomic testing, heard about it through “word of mouth”. Gibson, Hohmeier and Smith [52] found that 54% (*n* = 7) of participants had heard of pharmacogenomic testing, and other key terms associated with genomics, from the internet. Hahn et al. [53] used focus groups to discern participant understanding of the term ‘genomic medicine’, and found that some college students had heard of the term on the news and in biology classes.

Two studies used focus groups to discern genomic understanding [53, 58]. Mallow et al. [58] used a Community Participating Research approach. Community leaders suggested they use terms like ‘genes’ and ‘family health history’ rather than scientific terminology to assist discussions with the community. They found that participants were more likely to describe inheriting disease rather than inheriting health and wellness [58]. Hahn et al. [53] found that their focus group participants described ‘genomic medicine’ in terms of ‘genetics’, ‘family history’, ‘the genome project’, ‘using genetics to heal people’ and ‘cloning’. Ong et al. [60] also used focus groups to discuss baseline understanding of ‘personalised medicine’ and ‘precision medicine’ divided into the primary language spoken by the participants, allowing for discussions on terminology specific to the language.

Knowledge of genetic and/or genomic facts was directly assessed in two studies [46, 56]. Abdul Rahim et al. [46] and Khadir, Al-Qerem and Jarrar [56] both questioned respondents on their basic genetic literacy via survey questions. Abdul Rahim et al. [46] found that 56.1% of survey respondents (*n* = 464) were able to answer at least 5 out of 8 genetic literacy questions correctly, while Khadir, Al-Qerem and Jarrar [56] found that participants were knowledgeable in hereditary genetic information but not other scientific facts. Khadir, Al-Qerem and Jarrar [56] also gave participants the opportunity to self-report their knowledge of genetics. Many participants reported having ‘sufficient knowledge’ on basic medical uses of testing and potential social consequences, such as refusing testing and the rights of third parties to request genetic test results of individuals [56].

#### Question 2E: Who owns the IP generated by the technology?

For genomic testing, we have interpreted this question to mean whether patients own their genetic information or if it belongs to the group that conducts sequencing. Four studies found that participants had concerns about the privacy of their or their child’s genetic information [46, 53, 55, 57]. Hishiyama, Minari and Suganuma [54] also found that 37.1% (*n* = 1112) of their participants were concerned about management and storage of genetic information.

### Domain 3: Value Proposition

Greenhalgh et al. [39] use this domain to consider the value placed on the technology by healthcare professionals and the patient. Question 3B, demand-side value (to patient), is addressed in the primary sources.

#### Question 3B: Demand-side value (to patient)

Greenhalgh et al. [39] define this question as the downstream value of the technology, including the evidence of benefit to patients and affordability. Willingness to pay for genomic testing was directly assessed in three studies [50, 52, 57]. Gibson, Hohmeier and Smith [52] found that if the entire cost of the pharmacogenomic test was covered by insurance, 89% of participants (*n* = 24) would undertake testing [52]. Lee et al. [57] determined that age, gender, income, inconvenience of testing and prior knowledge all influenced whether participants would pay extra for personalised medical testing. Cost of testing was a concern for 44.8% of participants (*n* = 316) [57]. Edgar et al. [50] found that most adoptees (72.4%) and non-adoptees (80.3%) were willing to pay between US$1 and US$499. Education level was a predictor for adoptee willingness to pay, while income predicted willingness to pay in non-adoptees [50]. Abdul Rahim et al. [46] did not directly assess willingness to pay, however they noted that a high income was associated with participant willingness to partake in testing.

Hahn et al. [53] and Ong et al. [60] did not directly assess willingness to pay for genomic sequencing, but participants did express concerns about the cost of testing to the individual and whether there would be equitable access to testing.

### Domain 4: Adopter System

Greenhalgh et al. [39] use this domain to consider the adoption of the technology. The adopter system includes caregivers, healthcare professionals and patients. Question 4B addresses whether patients will adopt a technology, while 4C addresses if lay caregivers are available to facilitate adoption. As we did not include patients or lay caregivers in our review, we have adapted these definitions to incorporate hypothetical patients and/or carers under the term ‘genomic naive public’. Greenhalgh et al. [39] also emphasise patient acceptance and family conflict as influencing factors on use of technology.

Several personal values were identified across the included studies [46, 48–54, 56, 59]. Abdul Rahim et al. [46] and Hishiyama, Minari and Suganuma [54] found that contributing to science and medical research were reasons to partake [46, 54]. Other reasons for partaking in genomic testing suggested by Qatari adults included improved health knowledge and prevention of future health conditions [46]. This was also suggested by participants in Etchegary et al. [51], Hahn et al. [53] Khadir, Al-Qerem and Jarrar [56].

Bombard et al. [48] found that most of their participants preferred using scientific evidence (82%, *n* = 994) and receiving expert advice (74%, *n* = 897) when making important healthcare decisions. However, only half (53%) of participants had trust in healthcare (*n* = 639). Hahn et al. [53] also found that many participants were sceptical of genomic medicine specifically, and often associated it with genetic engineering and cloning despite these not being directly related to genomic testing. Some participants felt they did not need the information genomic testing could provide, while others who would hypothetically want testing, believed it could promote the development of new treatments and provide more information on family history [53].

Primary reasons for not willing to partake in testing, as noted by Abdul Rahim et al. [46] were lack of time, information or knowledge, and privacy concerns. Similar concerns were suggested by Hahn et al. [53] and Lee et al. [57]. Fear of the unknown was also suggested in Hahn et al. [53] and Mallow et al. [58]. Participants in Hahn et al. [53] also noted they may be uncomfortable with the results, and the results may be too deterministic.

Aside from general concerns about the nature of genomic testing, concern regarding communication of genetic information among family members was also highlighted [47, 51, 53, 56, 58, 61]. Ballard et al. [47] noted that most participants, whether asked to imagine either they or a family member had a genetic condition, believed other family members who might also be affected should be notified. Etchegary et al. [51] and Khadir, Al-Qerem and Jarrar [56] also found that most participants would share genomic test results with family members. Participants in Hahn et al. [53] generally had a positive view of learning about genetic information if it would help other family members as some had family members who had passed away without explanation. Mallow et al. [58], however, found that communicating genetic information to family members may be an issue. Participants cited several reasons for this including: upsetting children and the creation of family issues, older family members not willing to disclose information and stigmatisation by the community, particularly if the information in question regarded mental illness or substance abuse disorders [58]. Participants also suggested they would only discuss genetic risk if there was a health crisis in the family [58]. Etchegary et al. [51], although noting that many participants would want to share information, found that those with the highest education levels and income were less likely to share results with family members. Vermeulen et al. [61] also found that 17% of their participants (*n* = 160) were worried about “causing friction” within their families. However, participants who believed family history assessments were worthwhile cited disease prevention as a benefit to involving family members [61].

### Domain 6: Wider Context

Greenhalgh et al. [39] describe the wider context as the institutional and sociocultural contexts. Examples of the wider context include health policy, fiscal policy, statements and positions of professional and peak bodies, as well as law and regulation. Here, in order to respond to our research questions, we focus on the socio-cultural aspects of the public.

#### Question 6D: Socio-cultural context

Societal concerns were noted in many studies [51, 53–56, 58, 60, 61]. Twenty-two percent of participants (*n* = 1425) in the Hishiyama, Minari and Suganuma [54] study noted employment and insurance discrimination as a concern. This was also noted in Etchegary et al. [51] and Khadir, Al-Qerem and Jarrar [56]. Participants in Hahn et al. [53] and Mallow et al. [58] noted discrimination and segregation as key societal issues that may arise. One-third of participants (*n* = 311) in Vermeulen et al. [61] thought that individuals may be coerced into testing if it is normalised.

Cultural context may influence participant responses. For example, Abdul-Rahim et al. found the 45.1% of their respondents (*n* = 241) were in consanguineous relationships [46]. No other study reported on consanguinity, demonstrating that different cultures prioritise different elements when reporting. Abdul-Rahim et al. found that 70.9% population (*n* = 584) were willing to undergo genomic testing [46], whereas Dodson et al. found that 39.5% of their US population (*n* = 805) were somewhat interested and 19.1% (*n* = 389) were definitely interested in genomic testing [49]. These papers demonstrates that different cultures can influence perceptions of genomic testing. However, the Caucasian US population in Gibson et al. were more willing to undergo testing at 81.0% (*n* = 21) [52], showing that even within the same country there can be cultural differences that may lead to differences in perception.

## Discussion

In this study, we reviewed literature researching the genomic naive public’s perception of clinical genomics and clinical genomic testing. To our knowledge, this is the first review to do so. The NASSS framework developed by Greenhalgh et al. [39, 40] was used to identify and group concepts and themes across the included studies to form an overarching picture of public perceptions of genomics. We found that public perceptions could be applied to several NASSS domains. These included the domains regarding technology (Domain 2), value proposition (Domain 3), the adopter system (Domain 4) and the wider context (Domain 6). The NASSS framework provided a structured approach to organise results and identify the domains public perceptions can influence. Although the NASSS framework has been used previously to guide a systematic review [38] this is the first study, to our knowledge, to apply it to genomics research.

Our review demonstrates that public perceptions are not discrete, rather they underpin several aspects of the genomic technology development and implementation process, and that the public’s concerns are often far-reaching and insightful. These concerns include: management and storage of genomic information, privacy of genomic information, affordability of testing, scepticism and fear due to association of genetic and genomic testing with genetic engineering and cloning, employment and insurance discrimination, societal segregation, as well as the potential for family conflict due to genomic test results. These concerns range from the individual-level to the population-level.

### Domain 7 of the NASSS Framework: Adaptation over time

Another component of the NASSS framework not directly addressed in our results is Domain 7, ‘Embedding and adaptations over time’. Greenhalgh et al. [39] strongly suggest that their framework is reapplied after initial implementation of an innovative technology to determine if there are changes in the system to be addressed. This is particularly relevant for public perceptions which can be influenced by the ever-changing media discourse.

Evidenced through reports of Angelina Jolie’s prophylactic mastectomy in 2013, information communicated via mainstream media is particularly influential on public perceptions of genetic testing [62]. The more times individuals heard about Jolie’s story, the more over-confident they were in their perceived understanding of it [62]. This media story also led to confusion regarding the general population risk of breast cancer and when preventative surgery is advised [62]. Ballard et al. [47] also noted this phenomenon when the then UK Health Minister exaggerated the impact a prostate cancer polygenic risk score provided him, impacting the public perceptions. Hahn et al. [53] found that many participants often associated genomic medicine with genetic engineering and cloning, leading to increased sceptisim despite these being outside the scope of clinical genomic testing. The participants who recalled these media stories on genetic research described these as controversial [53]. In 2021, Horrow et al. [63] developed and validated the Genomic Orientation (GO) scale to determine attitudes towards genomic medicine. They suggest the use of this scale post-national events can identify any changes in attitudes [63]. Using a standardised scale across subgroups could also allow for systematic comparisons of perceptions. This exemplifies the importance of educating the public appropriately without the sensationalism mainstream media often brings.

### Challenges of diversity in genomic testing

Only three participant recruitment strategies were demonstrated across the included studies. The majority of studies attempted to recruit a representative sample, however there was no discernment of views from seldom heard groups. Three studies found their study population was higher educated than the general population [48, 59, 61] while others did not address this. This tendency suggests that the views found in these papers cannot be generalised to the whole population, but only represent those included.

Only one study in this review used targeted recruitment of a specific, underrepresented community (i.e. rural) [58]. Rural and regional populations were not separated out from the other studies. Views of Indigenous peoples and other underrepresented groups were also not discerned from the rest of the population in the included studies. Our findings suggest that there is a lack of research into seldom heard communities. However, they should be considered in implementation of genomic testing to ensure they do not experience further health disparities.

Lack of diversity in genetic and genomic research leads to reduced applicability of genomic medicine to those of non-Western European ancestry, with this overemphasis noted [14, 64, 65]. Landry et al. [64] noted across two genome databases that there was an over-representation of European ancestry across several types of disease. Accuracy of genetic information was also suggested as a concern in our findings [54, 58]. As recognised by the research community, accurate information regarding genomic testing is essential. Variant analysis of many ethnicities not only improves precision medicine for those of non-European ancestry and ensures equitable access to this technology, but also facilitates testing for the entire population as potential disease variants can be predicted more accurately [65]. Indigenous genomics poses additional concerns including culturally safe and appropriate research into collecting and analysing Indigenous genomes [66]. Indigenous peoples globally already face significant health disparities compared to the general population and with genomic technology advancing it is essential they are taken into consideration [66].

Diversity in participants is also important as families are often a product of their cultural context. Family dynamics were addressed in six studies [47, 51, 53, 56, 58, 61], suggesting that the structure of a family is highly influential, and therefore, the familial nature of genomic information cannot be understated. Not all individuals believe genomic testing is ‘helpful’ or will produce desirable information [67]. Therefore, wanting to deliver or receive genomic information from relatives may differ depending on a family’s cultural contexts. The British participant population in Ballard et al. [47] associated genomics with positive or neutral language, while the rural Virginian participant population in Mallow et al. [58] were more likely to associate genomics with disease, highlighting differences between Western populations. However, in Qatar where premarital screening is commonplace, there may be less resistance to genetic testing and involving family members [46]. Distrust in healthcare settings has been observed in Indigenous communities [68] and also in Bombard et al. [48], included in this review. Therefore, it is essential to consider the demographics, personal characteristics and cultural contexts of participants when analysing where there is resistance to genomic testing.

### Equity in genomic testing

Health equity has been defined by Peterson et al. [69] as “having the personal agency and fair access to resources and opportunities needed to achieve the best possible physical, emotional and social wellbeing” (pg. 741). However, participants in Hahn et al. [53] and Ong et al. [60] expressed concern about affordability, while three other studies noted income as a key influencing factor on willingness to undertake or pay extra for genomic testing [50, 52, 57]. Peterson et al. [69] note in their Health Equity Framework that the systems of power that determine individual and population access to health resources are a key influencing factor, and are noted by the public in the included studies.

Additional influencing factors on health equity include (a) relationships and networks, (b) individual factors and (c) physiological pathways [69]. Returning results to family members [47, 51, 53, 56, 58, 61] and potential societal segregation due to genomic testing causing further health disparities [61] are potential ways relationships and networks influence the equity of genomic testing. Physiological pathways that may influence health equity in genomic medicine include the ‘individuality’ suggested by pharmacogenomics and personalised medicine results. Genomic medicine is arguably most valuable to the public if it is equitable, and is demonstrated as concern of the public who were assessed in the included studies.

### Future research

Quantitative studies are often credited for their generalisability to the larger population [70]. For countries that have not conducted research in this area, it may be beneficial to ensure a representative population is recruited for an initial quantitative study. For example, Ballard et al. [47] used the service ‘YouGov’ to ensure their survey participant population was representative of the British population. Similar tools were also used in other included studies [49, 61]. We suggest the adaptation and use of the GO scale developed by Horrow et al. [63] to promote comparable results between countries, subpopulations etc. We also note that value proposition was only systematically assessed via willingness to pay studies, and that value should also be considered in terms of personal utility. Our review has already identified some of the key concerns expressed by different publics, which can be used to frame future semi-structured interviews or focus groups. Going more in depth into key concepts and allowing participants to describe genetics and genomics in their own language (i.e. without providing terminology) can promote a better understanding of the public’s perceptions. Qualitative studies can ensure the acknowledgement of the cultural contexts of the participant population, particularly in multicultural societies where it may otherwise be difficult to target these groups. This would also be useful in seldom heard communities e.g., rural communities, as demonstrated by Mallow et al. [58], and Indigenous communities, whose views are not separated out from the general participant population in the included studies. The NASSS framework could also be applied further by considering the interactions between domains at the jurisdictional level. For example, the current genomic testing regulations would be particularly useful in explaining trends in public perceptions. The NASSS framework can also be used to build a structured evidence-base for public perceptions of genomics testing.

### Limitations

This study allowed for the application of the NASSS framework, demonstrating how public perceptions can influence several domains for the implementation of innovative health technology. Limitations include the exclusion of non-English papers in this study, therefore some data may have been missed. We also excluded studies that focussed solely on genetics, without mention of genomics. Inconsistent terminology used across the studies may also lead to subjective interpretations of concepts, thereby limiting the analysis. However, the inconsistencies also demonstrate the importance of allowing the public to describe complex genomics concepts in their own vernacular.

## Conclusion

Although there has been extensive research into perceptions of various aspects of genetic testing, studies into public perceptions on clinical genomics is limited. This review consolidates the key concepts in this field thus far and structures them using the NASSS framework. Our study indicates the need for future research. Specifically, we recommend further quantitative studies using the GO scale developed by Horrow et al. [63] and qualitative studies to promote investigation into the views of seldom heard groups that are as yet under-represented in this field. To address this omission, targeted research to gather the views of for example, rural communities and Indigenous populations is required. This approach will ensure these groups are not disadvantaged as genomic medicine surges forward.

## Supplementary information

PRISMA-ScR Checklist

Search strategy

Full study characteristics

NASSS Application

Supplementary Files Reference List

## Data Availability

All data generated or analysed during this study are included in this published article and its supplementary information files.
