# Supplementary material for: How does the genomic naive public perceive whole genomic testing for health purposes? A scoping review
Source: Eur J Hum Genet. 2022 Oct 19;31(1):35–47. doi: 10.1038/s41431-022-01208-5 (PMC9822972; doi:10.1038/s41431-022-01208-5)
Supplement: Supplementary file 2 — Search strategy [file 41431_2022_1208_MOESM2_ESM.docx]

**Supplementary information 2:** Full search strategy

**Embase, Medline (OVID) and PubMed searched 21/09/2020 and 27/08/2021**

- #1 Title/Abstract
- (“public” OR “patient*” OR “societ*”) AND (“prefer*” OR “perception*” OR “understand*” OR “interpret*” OR “thought*” OR “opinion*” OR “view*” OR “knowledge” OR “prefer*” OR “expect*”)
- #2 Title/Abstract
- “genomic*” OR “exome-sequenc*” OR “genome-sequenc*” OR “sequence-analysis” OR “high-throughput-nucleotide-testing”
- #3 Title/Abstract
- “survey*” OR “questionnaire*” OR “interview*” OR “focus-group*”
- #4 All Fields
- NOTNLM OR publisher[sb] OR inprocess[sb] OR pubmednotmedline[sb] OR indatareview[sb] OR pubstatusaheadofprint
- #5 #1 AND #2 AND #3 AND #4
- #6 Title/Abstract
- “direct-to-consumer” OR “consumer-directed” OR “ancestr*” OR “genealog*” OR “recreation*” OR “personal-genomic-test*”
- #7 Title/Abstract
- (animal OR animals OR rat OR rats OR mouse OR mice OR rodent* OR murine OR sheep OR fly OR flies OR yeast OR drosophila OR nematode OR worm OR worms OR roundworm* OR round-worm* OR frog OR frogs OR xenopus OR zebrafish OR zebra-fish) NOT (human OR humans OR patient OR patients OR newborn* OR baby OR babies OR neonat* OR infan* OR toddler* OR pre-schooler* OR preschooler* OR kindergarten OR boy OR boys OR girl OR girls OR child OR children OR childhood OR adolescen* OR pediatric* OR paediatric* OR youth* OR teen OR teens OR teenage* OR school-aged* OR school-child* OR school-girl* OR school-boy* OR schoolgirl* OR schoolboy* OR man OR men OR woman OR women OR adult OR adults OR middle-age* OR elderly)
- #8 #5 NOT (#6 OR #7)
- Exclude: case reports or comment or editorial or guideline or letter or practice guideline
- Limited to: english language and yr="2010 -Current"
