# Supplementary material for: How does the genomic naive public perceive whole genomic testing for health purposes? A scoping review
Source: Eur J Hum Genet. 2022 Oct 19;31(1):35–47. doi: 10.1038/s41431-022-01208-5 (PMC9822972; doi:10.1038/s41431-022-01208-5)
Supplement: Supplementary file 3 — Full study characteristics [file 41431_2022_1208_MOESM3_ESM.docx]

**Supplementary File 3.** Full Study Characteristics

| Author | Country | Aim | Population type | Sample size (n) | Recruitment strategy | Representative population | Data collection strategy |
| --- | --- | --- | --- | --- | --- | --- | --- |
| Abdul Rahim et al (3) | Qatar | To measure public awareness and attitudes towards genetic and genomic testing in Qatari adults | - One-half of the respondents (49.2%) were male - Average age of respondents was ~36 years (range 18–75) - Over one-half (58.1%) of respondents were married - 45.1% of marriages were consanguineous - 89% of married respondents reported having children. - Almost 80% of the sample reported a secondary educational level or higher - approximately one-third of respondents (34.0%) reported a monthly household income of 70 000 QAR (19230 USD) or higher | 834 | Representative cell phone sample using a list-based dialling technique (Qatar has one of the highest cell phone penetration rates in the world) | Representative | Survey with questions regarding respondent demographics, knowledge, attitudes and willingness to undertake genomic testing. |
| Ballard et al (4) | UK | To evaluate opinions regarding genomic tests in general, if they have familial implications, and how incidental findings should be returned. | - 52% of respondents were female - 39.7% of respondents were aged 55+ - 57.4% of respondents were classed in social grades A, B and C1. (AB = higher and intermediate managerial, administrative, professional occupations. C1 = supervisory, clerical and junior managerial, administrative, professional occupations) - 38.7% of respondents work full time. | 1954 | Survey conducted via YouGov, a company who has access to a community of 6 million people worldwide. YouGov sent an email with a link to the survey to a sub-population (n = 2005) of their UK community of over 1 million respondents. | Representative | The survey questions aimed to ascertain how people conceptualise genomic tests in healthcare, in particular what they think regarding the potential of such tests to reveal unexpected information, or information of relevance to relatives of the person being tested. |
| Bombard et al (5) | Canada | To determine Canadians willingness to partake in expanded newborn screening compared to traditional newborn screening. | - 52% respondents were male - 40% of respondents were between the ages of 36-54 - 37% of respondents were from Ontario - 66% of respondents had at least one child - 82% of respondents had no known family history of genetic disease - 61% of respondents were married/had a partner under common law - 35% of respondents have a college education (reported as better educated than the general population) - 37% of respondents reported a household income of $40,000 to $79,999 (reported as high earners than the general population) | 1213 | Recruitment through an internet panel provided by Survey Sampling International (SSI), which hosts online panels to support market and academic research. A demographically diverse sample, reflective of the Canadian population by age, gender and region of residence, consistent with 2011 statistics Canada data were eligible to complete the questionnaire. | Not representative | Questionnaire included items to build and assess knowledge and to measure selected attitudes and demographics. The questionnaire included background information on the principles of screening and the varied effects of NBS (for example, early diagnosis and treatment, reproductive-risk information, false-positive results and overdiagnosis) followed by quizzes, a discrete choice experiment and a reasoning exercise. |
| Dodson et al (6) | USA | The aim of this study was to assess the baseline interest of the public in whole genome testing for oneself, parents’ interest in whole genome testing for their youngest children, and factors associated with such interest | - The majority of respondents reported themselves to be white, in good or better health, and not planning to have a child in the next 5 years - Education level, income, and political ideology were evenly distributed among parents and non-parents - Parents were more likely to be between ages 30 and 44 years, Hispanic, in at least good health, and planning to have a child in the next 5 years - Nonparents were more likely to be white and >60 years of age | 2144  Parents n=1539 (30.4%)  Non-parents n=605 (69.6%) | Cross-sectional internet-based sur- vey of a nationally representative sample of the US population. To ensure adequate representation, parents [defined as having child(ren) 0–17 years old living in the household] and particular racial minorities including African-Americans and Hispanics were oversampled. | Not representative | Survey with questions regarding respondent demographics, interest in whole genome sequencing for themselves and their youngest children. |
| Edgar et al (7) | USA | To have a better understanding of the specific genomic risk information adoptees are interested in learning | - Adoptees were more likely than non-adoptees to be older, female and to have children. - Adoptees were more likely to report having a condition they perceived to be genetic than non-adoptees (27.3% vs 13.1%, OR 2.5, 95%CI 1.4 to 4.4, p=0.002) and less likely to report a family history of a genetic condition (55.4% vs 86%, OR 0.2, 95%CI 0.12 to 0.33, p<0.001). - Adoptees reported prior genetic testing more often than non-adoptees and ancestry testing was the most commonly reported type of prior testing | Total = 341  Non-adoptees = 229  Adopted individuals = 112 | Non-adopted individuals were recruited via Amazon Mechanical Turk (MTurk), a crowdsourcing Internet platform. Adopted individuals were recruited through 14 organisations and Facebook groups for adoptees. | Non-adopted individuals were reported as representative due to the use of MTurk. Adopted individuals were not reported as representative. | Survey items included patient demographics, level of contact with biological relatives, knowledge of family history, interest in receiving elective genomic testing results, motivations for wanting testing, perceived utility of results, willingness to pay, and social and personal identity. |
| Etchegary et al (8) | Canada | To contribute to the gap in the literature and provide descriptive, attitudinal data that can inform the integration of genomics into clinical care in ways that accord with the public who will ultimately use the service | - 45 years old on average (range = 18–82 years) - Most were female and lived in the largest health authority, with smaller numbers from the more rural authorities. Most were married and had a least 1 child (mean = 1.3; SD = 1.2), a university degree, and annual incomes of more than USD 60,000 - Over 40% reported a genetic condition in their families, but very few reported having experience with genetic counselling (just over 12%) - Approximately 13% had used direct-to-consumer genomic services | 689 | Online survey was administered on SurveyMonkey and paid advertising enabled advertising of the survey link on Facebook. Facebook advertised the survey link to all registered users in the province. Targeted advertising was implemented as needed (e.g., to underrepresented users in the rural health authorities). The survey link was also shared widely through the research team’s and public council’s personal and professional networks, communication channels of Memorial University, and the provincial health authorities. | Not reported | 48-item survey that included of a mix of vignettes, scaled, open-ended, and demographic items. The survey began with an explanation of WGS and a summary of its potential risks and benefits. Survey items measured the following: (1) interest in WGS and information preferred for sequencing decisions, (2) interest in pharmacogenomic testing specifically, (3) attitudes toward various features of genome sequencing, (4) preferences for the return of incidental findings, (5) opinions about the secondary use of genomic data, and (6) demographic items. |
| Gibson et al (9) | USA | To determine patient knowledge, interest and willingness-to-pay for pharmacogenomics testing in a community pharmacy | - Response rate of 3.7% for those that opened the email - 100% were Caucasian - Mean age was 54.4 - 81% (n = 22) had private insurance - 19% (n = 5) had Medicare, Medicaid or public aid - The highest education level obtained for 44% (n = 12) was a bachelor’s degree - 26% (n = 7) attended some college or junior college - 26% (n = 7) obtaining a graduate degree - The annual household income was US$40,000–100,000 for 44% (n = 12) - 22% (n = 6) earned more than US$100,000 | 27 | Qual­trics survey was distributed to 7019 email addresses of patients and customers of an independent community pharmacy.  The study site is a community pharmacy in Murfreesboro (TN, USA) that offers a wide variety of clinical services, including bioidentical hormone replacement therapy consultations, an immunization travel clinic, medication compounding, diabetes education classes and prescription delivery. | Not representative but not reported | Survey with four main parts: 1) patient demographics  2) patient pharmacogenomics knowledge 3) patient interest in pharmacogenomic testing 4) willingness-to-pay for a pharmacogenomic testing service |
| Hahn et al (10) | USA | The Genomedical ConnectionTM is a demonstration project aimed at developing a model to incorporate genomic medicine into community health care….  This paper ad- dresses the first study done as part of the educational needs assessment for the community. Using focus group methodology, we assessed the community’s awareness and perception of genomic medicine and preferences regarding educational strategies and content.  Baseline knowledge will be used to inform educational interventions. | - 47% male - 62% non-Hispanic Whites - 35% African American - 3% Hispanic or Asian. - Participants ranged from undergraduates to seniors - Perceived group demographics approximated the community’s demographics: 45% of residents were male, 65% were non-Hispanic Whites, 32% were African American, 5% were Hispanic, and 1% were Asian.   Occupations:   - School teacher n=14 - College students n=48 - Seniors (retired adults over the age of 55) n=21 - Veterans n=6 - Active military n=32 | 121 | County Health Department and Chamber of Commerce representatives were used as key informants to identify organizations within the County that provided a representative population in terms of gender, ethnicity, religion, age, and socioeconomic status. Churches, colleges, employers, social groups, local Veteran’s Administration, and Army National Guard units were contacted for inclusion. The study participants were recruited from a single urbanized community in the Southeastern United States whose collective perceptions cannot be projected onto other communities. Also, the sample was limited to English-speaking participants who were self-selected. No effort was made to divide participants by age, race, or gender, although they were separated by rank at the Army Reserve Unit. | Representative for gender and ethnicity only | To assess the community’s awareness and perception of genomic medicine and preferences regarding educational strategies and content, an open-ended, semi-structured interview guide was produced based on input from genetic counselors, researchers, community health educators, and community health nurse researchers. This information was combined with reported population data to produce the script. |
| Hishiyama et al (11) | Japan | This study aims to elucidate the public attitude towards the handling of genetic information during research and general medicine in the Japanese adult population | Authors recruited 300 males and 300 females for each age group (20-29; 30-39; 40-49; 50-59; 60-69 years of age). | 3000 | The participants of the survey were 3000 people recruited from the Ordinary Citizens Panel consisting of people aged 20 to 69 years. The Panel belongs to the “Ordinary Citizens Market Forecasting System” managed by the Mitsubishi Research Institute. | Not representative but not reported | Survey with questions regarding respondent demographics, knowledge and perception of genetic information, preferences when receiving results, attitudes towards the handling of genetic information, and concerns about genetic testing. |
| Joseph et al (12) | USA | To inform policy debates by examining the views, perspectives, and values of healthy pregnant women, and parents of children with primary immunodeficiency disorders about both traditional newborn screening and expanded newborn screening | - Mean age = 31.8 - 59% were born in the United States. - 12 participants self-identified as ‘white’ - 6 participants self-identified as ‘Black/African American’ - 6 self-identified as ‘Other’ - 8 participants had some college education - 7 participants were high school graduates - 7 participants held a graduate degree. - 13 participants had an annual household income of less than $20,000 - 10 participants earned more than $100,000 | 31  Socioeconomically and ethnically diverse pregnant women (n=26)  Parents of children diagnosed with a primary immunodeficiency disorder (n=5) | Pregnant women were receiving prenatal care at either of two urban California medical sites, an academic medical hospital (AMC) and a public hospital (PH). | Not representative, however not expected to be representative | Focus groups to discuss initial impressions and concerns regarding the Californian NBS program. Participants were also provided with two case studies of expanded NBS to facilitate further discussion. |
| Khadir et al (13) | Jordan | To assess the knowledge and attitude towards genetic testing of the Jordanian population in general. It analyses the association between knowledge, attitude and several demographic fac- tors. In addition, since genetic testing is also needed to confirm clinical diagnosis of several immune diseases, this study also evaluates the knowledge and attitude of patients with immune diseases and how it compares to that of the general Jordanian population | - Majority (72.1%) were females - One-third of them were between 18 and 29 years (29.9%) - One-third were between 30 and 39 years (30.2%) - Most of them were married (64.2%) - More than half of the participants (60.8%) had a bachelor’s degree - 27.5% were postgraduates - <1% had only primary education - About one fifth of the participants were studying/working in a health-related field (18.3%) - The participants’ household average monthly income varied from <300 JD (22.6%) to more than 2000 JD (11.3%). - Most of the participants were healthy (74.4% had no immune diseases) - 13.1% had hypersensitivity disorders - 9.1% had an autoimmune disease - More than half of them (51.8%) had a first degree relative with a chronic or autoimmune disease - Almost two-thirds of the participants (66.1%) never had information about genetics and genetic testing | 1149 | The online questionnaire was distributed using different generic social media plat- forms dedicated for Jordan in addition to other platforms targeting different Jordanian cities to ensure better representation of different segments of the Jordanian population. | Not reported | Survey questions include: demographic characteristics, genetic knowledge, perceived knowledge of genetics and attitudes toward genetic testing. |
| Lee et al (14) | Korea | To evaluate the awareness and attitude towards personalised medicine in Korean adults | - Survey participants included more women (58.6%) than men (41.4%) - Participants were approximately evenly distributed by 10-year age groups. - Sixty-eight percent of respondents had college or post-graduate education - About two-thirds of respondents exhibited a positive view concerning the performance of NHI in public health improvement | 703 | Survey was distributed to 706 adults who visited community pharmacies or public healthcare centers between December 31, 2012 and January 14, 2013.  13 study sites featuring diverse demographic and medical characteristics of respondents were chosen. Study sites were comprised of four pharmacies that primarily fill prescriptions for outpatients from general hospitals, seven community pharmacies providing over-the-counter medications and filling prescriptions from nearby local clinics, and two community healthcare centers. All sites were located in the Seoul metropolitan area. | Not reported | Developed a questionnaire to examine public awareness and several aspects of attitudes regarding PM using PGx information. To identify any ambiguity in questions, we conducted a pilot test prior to completing the final version of the questionnaire which comprised three domains: public knowledge/awareness of personalized medicine, public attitude toward personalized medicine, and public acceptance of integrated pharmacogenomic testing as part of the national health examination. |
| Mallow et al (15) | USA | To assess genomic and epigenetic knowledge and beliefs in rural West Virginia, USA | - Age of respondents ranged from 21 to 81 years - Most respondents had a household income of less than $40,000, were female and most were married, completed at least a HS/GED or some college education working either part-time or full-time. | Survey: n=68  Focus group: n=93 | The recruitment for each forum was directed by the Community Partnership Board and the research team. Approximately 100 personal invitations per community were sent to community leaders and lay persons 1–3 weeks in advance. In addition, flyers were hung in local areas (places of worship, grocery stores, community centers, etc.)  The survey was distributed to focus group participants at the end of the session. | Not reported | Focus groups began with a family health story from a respected leader in the community. The community led the discussion and focus groups usually lasted from 1.5 to 2 hours. Discussions were around genes and family health history.  The survey contained basic demographic information as well as qualitative and quantitative questions on their knowledge of family health history, willingness to partake in genetic studies, and the perceived influence of environmental and lifestyle factors on hereditary condition. |
| Okita et al (16) | Japan | To survey Japanese adults interests and concerns of whole genome testing, their willingness to partake in research, and the factors that influence their attitudes | - 1196 males (49.9%) and 1203 females (50.1%) - Mean age of 44.91 years - 29.6% of respondents ended their education with high school compared to 46.5% of people in the 2010 national census - 44.5% of our respondents had graduated from either university or graduate school compared to 19.9% of people in the census data - The 2015 national census showed that approximately 16.7% (20 million) people had part-time or temporary employment, while approximately 27.5% (33 million) had permanent employment. In our survey, 17.3% of respondents were non- regularly employed and 36.0% had regular employment - On average there were 3 people in a household. - 54.6% of participants were married, 38.6% were unmarried, 6.8% were divorced - 14.0% earned less than 2,000,000 JPY, 14.1% earned 3,000,000-4,000,000 JPY, 14.1% earned 4,000,000-5,000,000 JPY, 9.7% earned greater than or equal to 10,000,000 JPY | 2399 | Participants were registered as part-time survey assistants with Video Research Ltd., to which we outsourced part of the survey.  The subjects comprised males and females aged 16 years or older who matched the age distribution of the Japanese population based on the Japanese government’s national population census in 2010 | Not representative | A survey of awareness around WGS and studies using WGS  Prior to each section where respondents choose their answers, we briefly explained WGS and WGS study-related issues, including an out- line of WGS studies, data sharing, and return of results, to the respondents to provide them with the minimum knowledge required to answer the questionnaire. |
| Ong et al (17) | Singapore | To determine whether the Singapore population prefer the term ‘precision medicine’ or ‘personalised medicine’. | - The participants were relatively well-educated with just over half holding a degree or post graduate qualification (13/ 24). - The ages of participants ranged from 21 to ≥ 58 years. - Research population was relatively highly educated (54.2% had a degree or post graduate qualification), young (most were under 37 years old), and some were familiar with and evidently interested in participating in research. Nation- ally, the proportion of Singaporean residents with an undergraduate degree or higher is 31.6% (Singapore Department of Statistics 2019). Effort was taken to ensure a wide spread of ages in our focus groups, but ensuring a nationally representative sampling was not the aim. | Total = 24  English-speaking = 11  Mandarin-speaking = 8  Malay-speaking = 5 | Not reported | Not representative | Focus groups began with discussions on baseline understanding of ‘precision medicine’ and ‘personalised medicine’. After an education video, participants were asked again which term they preferred. Participants were then able to discuss other concerns or thoughts the video provoked. |
| Vermeulen et al (18) | The Netherlands | To the Dutch public’s opinion towards preventive genomics, their attitudes towards genetic testing and family history-based risk assessment for common chronic conditions, and what influences these attitudes. | - The mean age of all respondents was 59.1 (range 18–91) years - Mean ages were 62.7 (range 18–91) years and 56.5 (range 18–91) years for men and women, respectively - Younger respondents were more often higher educated (P< 0.001) - Compared with the Dutch population, the respondents were more often female, older, and higher educated. | 978 | Consumer panel representative of the Dutch population | Not representative | Survey with questions regarding respondent demographics, knowledge of genetic tests, purchase of commercial genetic tests, |

NBS: newborn screening.
