# Supplementary material for: How does the genomic naive public perceive whole genomic testing for health purposes? A scoping review
Source: Eur J Hum Genet. 2022 Oct 19;31(1):35–47. doi: 10.1038/s41431-022-01208-5 (PMC9822972; doi:10.1038/s41431-022-01208-5)
Supplement: Supplementary file 4 — NASSS Application [file 41431_2022_1208_MOESM4_ESM.docx]

**Supplementary File 4.** Application of nonadoption, abandonment, scale-up, spread, and sustainability (NASSS) framework (2) domains to public perceptions of genomic naïve public

| **Framework component (2)** | **Definition (2)** | **Adapted definition for application in practice** | **Applied to Public perceptions** |
| --- | --- | --- | --- |
| **Domain 2: Technology** | | | |
| **Question 2B:**  Type of data generated | “Question 2B considers the knowledge generated or made visible by technology. This includes not only the accuracy of the data but also the extent to which those data are accepted, trusted, and considered sufficient for decision making. Engaging with the data generated by patient-facing technologies may inform, educate, and empower patients and lay caregivers. But data may also be misinterpreted by the patient or cause distress.” | We did not adapt the definition provided by Greenhalgh et al. | Hishiyama et al (11) and Mallow et al (15) found that accuracy of genetic information was a concern for their participants. |
| **Question 2C:** Knowledge needed to use the technology | “Question 2C addresses the knowledge and support needed to use the technology. Some technologies are much easier to operate than others; some require frequent troubleshooting; and some assume a different organizational role—or even an altered professional identity—for the user. Some patient-facing technologies require no knowledge form the patient; others require clinical knowledge, technical knowledge, and the ability to make judgments about (for example) what counts as urgent.” | Rather than considering the clinical technicalities of genomic sequencing, here we consider baseline lay person knowledge on the testing and how this might influence their hypothetical decision to undertake testing. | If the public have heard about various genomic terms:   - Abdul-Rahim et al (3) found that a third of their participants had heard of genomic testing, while at least half had heard of genetic testing - Gibson et al (9) found that 54% (n=7) of participants had heard of pharmacogenomic testing - Hahn et al (10) found that most participants had not heard of genomic testing or personalised medicine - Hishiyama et al (11) found that more than two-thirds of participants had heard of genetic-related terminology while fewer participants had heard of newer, genomics-related terminology - Ong et al (17) found that English-speaking and Mandarin-speaking participants had heard of the term ‘personalised medicine’ but not ‘precision medicine’. Malay-speaking participants had not heard of either   How the public have heard about genomics (e.g. word of mouth, internet, news):   - Abdul-Rahim et al (3) found that 30% of their participants who had heard about genomic testing found out about it through 'word of mouth’ - Hahn et al (10) found that some college students had heard of genomic medicine through the news or in biology classes   How participants describe genomics:   - Mallow et al (15) found that participants used language about inheriting illness rather than health - Hahn et al (10) found that participants were likely to explain genomic medicine in terms of genetics, family history and genetic cloning   Genetic literacy:   - Abdul-Rahim et al (3) found that 56% of their respondents were able to answer at least 5 out of 8 genetic literacy questions correctly - Khadir et al (13) found that participants were knowledgeable in hereditary genetic information but not other scientific facts   Many of Khadir et al’s (13) respondents reported they had sufficient knowledge on basic medical uses of genetic testing and social consequences of testing |
| **Question 2E:**  Who owns the IP generate by the technology? | No definition provided in Greenhalgh et al (2). However, definition provided by Greenhalgh (17). IP ownership can either be considered ‘unambiguous and agreed’ upon, or if the technology generates higher-order data then IP may be more complex. | In terms of genomic sequencing, we have interpreted this as whether patients own their own genomic or if it belongs to the researcher/clinic conducting the testing and how hypothetical patients may perceive this. | Abdul-Rahim et al (3), Hahn et al (10) and Lee et al (134) found that participants had privacy concerns.  Joseph et al (12) found that privacy of newborn genomic information was a key concern.  Hishiyama et al (11) found that management and storage of genetic information was a concern. |
| **Domain 3: Value proposition** | | | |
| **Question 3B:**  Demand-side value (to patient) | “Question 3B addresses downstream value, which follows the demand-side logic of health technology appraisal, reimbursement, and procurement (ie, relates to evidence of benefit to patients and real-world affordability)” | We focus on patients’ ability to afford genomic sequencing and their willingness to pay as a proxy for perceived value of sequencing. | Edgar et al (7), Gibson et al 9) and Lee et al 14) assessed willingness to pay for genomic testing.  Monetary value explored in an indirect manner in Abdul-Rahim et al (3), who found that respondents with a high income were more willing to partake in testing.  Hahn et al (10) and Ong et al (17) found that participants were worried about the cost to the individual and equitable access. |
| **Domain 4: Adopter System** | | | |
| **Question 4B:**  Patient (passive v active input) | “Question 4B addresses adoption by patients or clients, including acceptance (hence symbolic meaning and aesthetics) and the work required of them.” | 4B and 4C were combined as we did not include studies that had genomics/genetics patients. Therefore, all ‘patients’ and ‘carers’ here are hypothetical and can be classified as the ‘genomic naïve public’. We considered various factors respondents thought may influence their hypothetical decision to partake in genomic sequencing. | Most studies gathered insight into the personal values and beliefs which would hypothetically lead to the use of genomic testing (3, 5-11, 13).  Personal values include:   - Wanting to contribute to science and medical research - Improved personal health knowledge vs fear of the unknown/not needing to know/determinism - Prevention of future health conditions - Wanting to know more about family history of disease - Mistrust in government/scientists (5, 10)   Six studies mention family dynamics.  Studies that showed that participants thought other family members should be notified of results include Hahn et al (10), Khadir et al (13), Etchegary et al (8) and Ballard et al (4). Mallow et al (5) and Vermeulen et al (18) found that participants were concerned about causing friction within their families. |
| **Question 4C:**  Carers (available, type of input) | “Question 4C addresses the assumptions that may be built into the technology (or the linked service model) about the availability and behavior of lay caregivers. We encountered many cases of nonuse of all patient-facing technologies that were explained by weak or absent social networks, limited information technology skills (and distrust of technology) among lay caregivers, or long-standing family conflicts, which the technology sometimes brought to the surface but never solved…” |  |  |
| **Domain 6: Wider context** | | | |
| **Question 6D:**  Socio-cultural context | “Question 6A relates to the wider institutional and sociocultural context, which in our case studies was often key to explaining an organization’s failure to move from a successful demonstration project (heavily dependent on particular champions and informal workarounds) to a fully mainstreamed service (scale-up) that was widely transferable (spread) and that persisted long term (sustainability). Aspects of wider context that proved pivotal in our case studies included health policy (including which service models were formally approved for funding—see example in Multimedia Appendix 2 of the difficulties in securing a nationally approved tariff for remote consultations), fiscal policy (the overall amount of funding available locally and nationally for health and care provision), the position taken by professional bodies and defense societies (who de facto defined what was acceptable professional practice), and legal and regulatory aspects of patient-facing technology development.” | Rather than just considering personal values as in Domain 4C, here we also included wider societal concerns expressed by participants/respondents. As we focus on lay people in this review, we did not consider any government positions on genomic sequencing as it is beyond our scope. We did, however, consider familial and cultural dynamics and their impact on the individual. | Various societal concerns were noted in several of the studies (8, 10-13, 15, 17, 18). Some of these concerns included:   - Employment discrimination - Insurance discrimination - Societal segregation and discrimination - Coercion into genomic testing (i.e. loss of autonomy) (18) |
