# Supplementary material for: How does the genomic naive public perceive whole genomic testing for health purposes? A scoping review
Source: Eur J Hum Genet. 2022 Oct 19;31(1):35–47. doi: 10.1038/s41431-022-01208-5 (PMC9822972; doi:10.1038/s41431-022-01208-5)
Supplement: Supplementary file 5 — Supplementary Files Reference List [file 41431_2022_1208_MOESM5_ESM.docx]

**Supplementary Files Reference List**

1. Tricco AC, Lillie E, Zarin W, O'Brien KK, Colquhoun H, Levac D, et al. PRISMA extension for scoping reviews (PRISMA-ScR): checklist and explanation. Annals of internal medicine. 2018;169(7):467-73.

2. Greenhalgh T, Wherton J, Papoutsi C, Lynch J, Hughes G, Hinder S, et al. Beyond adoption: a new framework for theorizing and evaluating nonadoption, abandonment, and challenges to the scale-up, spread, and sustainability of health and care technologies. Journal of medical Internet research. 2017;19(11):e8775.

3. Abdul Rahim HF, Ismail SI, Hassan A, Fadl T, Khaled SM, Shockley B, et al. Willingness to participate in genome testing: a survey of public attitudes from Qatar. J Hum Genet. 2020;65(12):1067-73.

4. Ballard LM, Horton RH, Fenwick A, Lucassen AM. Genome sequencing in healthcare: understanding the UK general public's views and implications for clinical practice. Eur J Hum Genet. 2020;28(2):155-64.

5. Bombard Y, Miller FA, Hayeems RZ, Barg C, Cressman C, Carroll JC, et al. Public views on participating in newborn screening using genome sequencing. European Journal of Human Genetics. 2014;22(11):1248-54.

6. Dodson DS, Goldenberg AJ, Davis MM, Singer DC, Tarini BA. Parent and public interest in whole-genome sequencing. Public Health Genomics. 2015;18(3):151-9.

7. Edgar J, Bao A, Maga T, Schwartz M, Yates C, Spencer S. Adopted individuals’ interest in elective genomic testing. Journal of Medical Genetics. 2022;59(2):197.

8. Etchegary H, Pullman D, Simmonds C, Rabie Z, Rahman P. Identifying Aspects of Public Attitudes Toward Whole Genome Sequencing to Inform the Integration of Genomics into Care. Public Health Genomics. 2021:1-12.

9. Gibson ML, Hohmeier KC, Smith CT. Pharmacogenomics testing in a community pharmacy: patient perceptions and willingness-to-pay. Pharmacogenomics. 2017;18(3):227-33.

10. Hahn S, Letvak S, Powell K, Christianson C, Wallace D, Speer M, et al. A community’s awareness and perceptions of genomic medicine. Public Health Genomics. 2010;13(2):63-71.

11. Hishiyama Y, Minari J, Suganuma N. The survey of public perception and general knowledge of genomic research and medicine in Japan conducted by the Japan Agency for Medical Research and Development. Journal of Human Genetics. 2019;64(5):397-407.

12. Joseph G, Chen F, Harris-Wai J, Puck JM, Young C, Koenig BA. Parental Views on Expanded Newborn Screening Using Whole-Genome Sequencing. Pediatrics. 2016;137:S36-46.

13. Khdair SI, Al-Qerem W, Jarrar W. Knowledge and attitudes regarding genetic testing among Jordanians: An approach towards genomic medicine. Saudi Journal of Biological Sciences. 2021;28(7):3989-99.

14. Lee IH, Kang HY, Suh HS, Lee S, Oh ES, Jeong H. Awareness and attitude of the public toward personalized medicine in Korea. PLoS ONE [Electronic Resource]. 2018;13(2):e0192856.

15. Mallow JA, Theeke LA, Crawford P, Prendergast E, Conner C, Richards T, et al. Understanding Genomic Knowledge in Rural Appalachia: The West Virginia Genome Community Project. Online J Rural Nurs Health Care. 2016;16(1):3-22.

16. Okita T, Ohashi N, Kabata D, Shintani A, Kato K. Public attitudes in Japan toward participation in whole genome sequencing studies. Hum Genomics. 2018;12(1):21.

17. Ong S, Ling J, Ballantyne A, Lysaght T, Xafis V. Perceptions of ‘Precision’ and ‘Personalised’ Medicine in Singapore and Associated Ethical Issues. Asian Bioethics Review. 2021;13(2):179-94.

18. Vermeulen E, Henneman L, van El CG, Cornel MC. Public attitudes towards preventive genomics and personal interest in genetic testing to prevent disease: a survey study. European Journal of Public Health. 2014;24(5):768-75.

19. Greenhalgh T, Abimbola S. The NASSS framework-a synthesis of multiple theories of technology implementation. Stud Health Technol Inform. 2019;263:193-204.
